# LncRNA ANRIL mediates endothelial dysfunction through BDNF downregulation in chronic kidney disease

**DOI:** 10.1038/s41419-022-05068-1

**Published:** 2022-07-29

**Authors:** Hong Su, Bing Liu, Huimin Chen, Tingwei Zhang, Tongtong Huang, Yue Liu, Cheng Wang, Qiqi Ma, Qianhui Wang, Zhimei Lv, Rong Wang

**Affiliations:** 1 Department of Nephrology, Shandong Provincial Hospital Affiliated to Shandong First Medical University, 250021 Jinan, Shandong China; 2 Department of Nephrology, Shandong Provincial Hospital, Cheeloo College of Medicine, Shandong University, 250021 Jinan, Shandong China

**Keywords:** Chronic kidney disease, Vascular diseases

## Abstract

Endothelial dysfunction is common in patients with chronic kidney disease (CKD), but the mechanism is unknown. In this study, we found that the circulating ANRIL level was increased and correlated with vascular endothelial dysfunction in patients with CKD, also negatively correlated with plasma brain-derived neurotrophic factor (BDNF) concentration. We constructed the ANRIL knockout mice model, and found that ANRIL deficiency reversed the abnormal expression of BDNF, along with endothelial nitric oxide synthase (eNOS), vascular adhesion molecule 1 (VCAM-1) and Von Willebrand factor (vWF). Meanwhile, mitochondrial dynamics-related proteins, Dynamin-related protein 1 (Drp1) and mitofusins (Mfn2) level were also recovered. In addition, in vitro, serum derived from CKD patients and uremia toxins induced abnormal expression of ANRIL. By making use of the gain- and loss-of-function approaches, we observed that ANRIL mediated endothelial dysfunction through BDNF downregulation. To explore the specific mechanism, RNA pull-down and RNA-binding protein immunoprecipitation (RIP) were used to explore the binding of ANRIL to histone methyltransferase Enhancer of zeste homolog 2 (EZH2). Further experiments found increased EZH2 and histone H3 lysine 27 trimethylation (H3K27me3) levels at the BDNF promoter region. Collectively, we demonstrated that ANRIL mediate BDNF transcriptional suppression through recruitment of EZH2 to the BDNF promoter region, then regulated the proteins expression related to endothelial function and mitochondrial dynamics. This study provides new insights for the study of endothelial dysfunction in CKD.

## Introduction

Chronic kidney disease (CKD) is a public health problem with an estimated global prevalence of 13.4% [1], which is also epidemiologically characterized by a high mortality rate [2]. Cardiovascular disease is the main cause of death in patients with CKD [3], and with the decline of renal function, the incidence of cardiovascular diseases such as atherosclerosis increased significantly [4, 5]. Endothelial dysfunction, encompassing inappropriate regulation of vascular tone, activated inflammatory response and impaired antithrombogenic property, is now regarded as one of the earliest phenomena of cardiovascular diseases [6, 7]. While in CKD, problems as toxin accumulation, inflammation, calcium and phosphorus metabolism disorders, can disrupt endothelial homeostasis and cause endothelial dysfunction [8], which also play a key role in the occurrence of cardiovascular complications, however, the underlying mechanisms remain unclear.

Antisense non-coding RNA in the INK4 locus (ANRIL) is a long non-coding RNA (lncRNA) located in the antisense direction of the INK4B-ARF-INK4A motif cluster of 9p21 chromosomes, and its gene single nucleotide polymorphism is associated with a variety of atherosclerotic vascular diseases such as coronary heart disease and myocardial infarction [9]. Studies have shown that its expression was associated with risk for coronary atherosclerosis, carotid arteriosclerosis peripheral artery disease, and other vascular disease [10]. Meanwhile, the upregulated expression of ANRIL can also be detected in coronary atherosclerotic plaques [11], suggesting that ANRIL plays an important role in cardiovascular diseases. ANRIL is widely expressed in human vascular endothelial cells. Recent data demonstrated that in patients starting on hemodialysis, ANRIL polymorphisms could identify risk of major adverse cardiovascular event [12] which indicated that ANRIL played an important role in CKD and was involved in the occurrence and development of cardiovascular complications in CKD. However, the role and mechanism of ANRIL in cardiovascular complications are still unclear.

Brain-derived neurotrophic factor (BDNF) may be a new important predictor of cardiovascular disease. It has been reported that the level of circulating BDNF decreased in angina [13] and coronary artery disease [14, 15]. Consistently, a prospective analysis in a large community-based cohort demonstrating that higher serum BDNF levels are associated with a decreased risk for future cardiovascular disease events and mortality [16]. Studies demonstrated that BDNF and its receptors are also highly expressed in heart and aorta, the expression being prominent in endothelial cells in which its expression is dependent on endothelial function [17]. Also, BDNF in the endothelium participates in maintaining endothelial integrity and stimulating angiogenesis [18, 19]. Additionally, studies have found that BDNF was also significantly reduced in plasma of patients with end-stage renal disease [20], suggesting that BNDF may play an important role in vascular disease in CKD. Therefore, whether ANRIL can mediate endothelial dysfunction by BDNF regulation remains to be elucidated.

In the present study, we provided the evidence that ANRIL were associated with endothelial dysfunction in CKD, then the inducing factors and specific mechanism were explored. This may provide a new theoretical basis for the occurrence and development of endothelial dysfunction in CKD.

## Results

### LncRNA ANRIL was inversely correlated with renal function and endothelial function

**Fig. 1:**
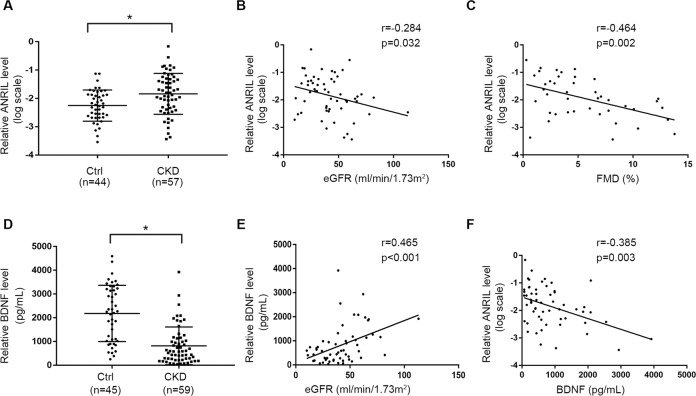
LncRNA ANRIL levels were inversely correlated with endothelial function and BDNF concentration. **A** ANRIL levels were elevated in circulation of patients with CKD. **B** Correlation analysis showed a negative correlation between eGFR and ANRIL levels (*n* = 57). **C** Correlation analysis showed a negative correlation between FMD detection values and ANRIL levels in patients with CKD (*n* = 42). **D** BDNF concentration were decreased in circulation of patients with CKD. **E** Correlation analysis showed a positive correlation between eGFR and BDNF concentration (*n* = 59). **F** Correlation analysis showed a negative correlation between BDNF concentration and ANRIL levels in patients with CKD (*n* = 57). Data were shown as mean ± SD. (Student’s *t* test, Mann–Whitney test, Spearman’s correlation analysis. ^*^P < 0.05 versus control group).

Baseline demographic and clinical characteristics of all patients are shown in Supplementary Table 1. Notably, other than the presence or absence of CKD and Blood Pressure, the two groups were comparable in sex, age, and other variables that might affect ANRIL levels (e.g. existing coronary artery disease, diabetes mellitus, and lipid profiles). RNA was extracted from clinical plasma samples to detect the content of lncRNA ANRIL. The results showed that the plasma ANRIL level of CKD patients were significantly higher than that of the healthy group (Fig. 1A). And, the plasma level of ANRIL was negatively correlated with estimated glomerular filtration rate (eGFR) (*r* = −0.284, *p* = 0.032) (Fig. 1B)

To examine whether circulating ANRIL level was associated with endothelial function, noninvasive evaluation of vascular endothelial function was conducted by Flow-mediated dilatation (FMD) test with Doppler ultrasound. As shown in Supplementary Table 1, CKD patients showed a lower FMD level (5.52% vs 7.79%), and the FMD detection values in patients with CKD showed a negative correlation with ANRIL level (*r* = −0.464, *p* = 0.002) (Fig. 1C).

### BDNF concentration was inversely correlated with renal function and ANRIL level

Further, plasma BDNF concentration was detected. The result showed that plasma BDNF concentration was also lower in patients with CKD (813.94 pg/mL vs 2179.06 pg/mL) (Fig. 1D), and correlated with eGFR positively (*r* = 0.465, *p* < 0.001) (Fig. 1E). Moreover, the BDNF concentration in patients with CKD showed a negative correlation with ANRIL level (*r* = −0.385, *p* = 0.003) (Fig. 1F). These suggested that ANRIL may be associated with endothelial dysfunction in patients with CKD, and BDNF could play an important role in this process.

### Inhibition of ANRIL alleviated endothelial dysfunction in CKD mice models

**Fig. 2:**
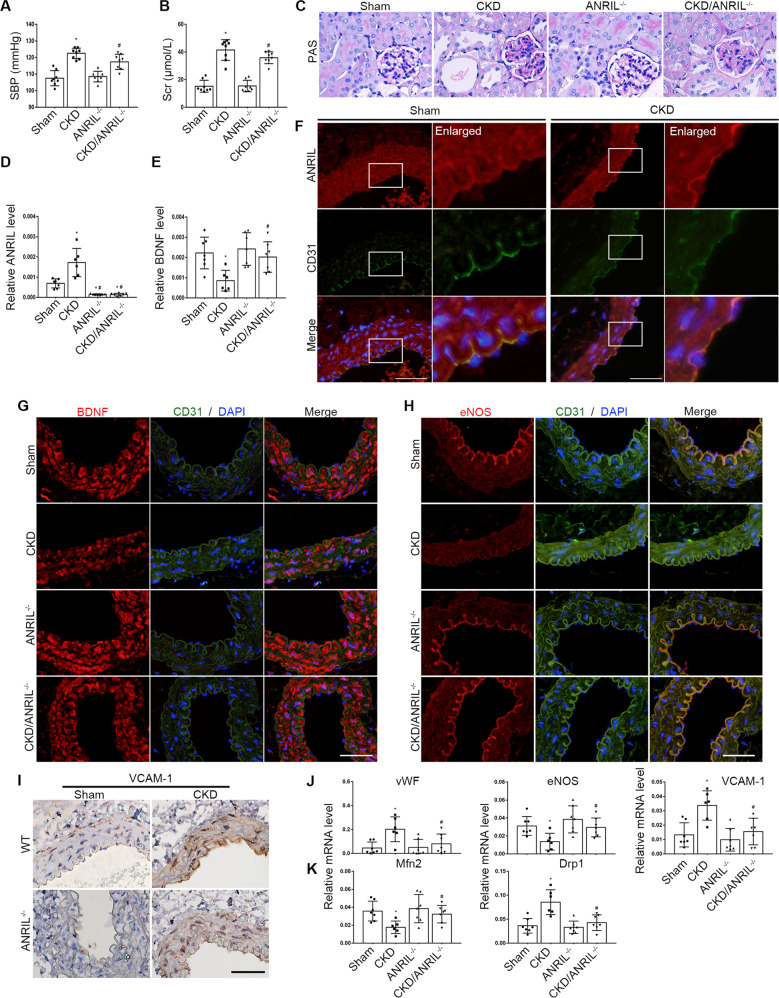
Inhibition of ANRIL alleviated endothelial dysfunction in CKD mice models. **A** Quantification of systolic pressure (SBP) in different groups of mice (*n* = 8). **B** Serum creatinine (Scr) concentration from different groups of mice (*n* = 8). **C** Periodic Acid-Schiff staining was used to assess the histological changes of kidney. Scale bars = 50μm. **D** The expression level of ANRIL in abdominal aortas was detected by realtime PCR (*n* = 6–7). **E** mRNA levels of BDNF in abdominal aortas was detected by realtime PCR (*n* = 6–7). **F** Expression and distribution of ANRIL in abdominal aortas were detected by FISH combined with CD31 immunofluorescence double staining. Scale bars = 50 μm. **G** Immunofluorescence staining for BDNF in aortas from different groups of mice. Scale bars = 50 μm. **H** Immunofluorescence staining for eNOS in aortas of each group. Scale bars = 50 μm. **I** Immunohistochemical staining for VCAM-1 in aortas f of each group. Scale bars = 50μm. **J** mRNA levels of vWF, eNOS, VCAM-1 in abdominal aortas was detected by realtime PCR (*n* = 6–7). **K** mRNA levels of Mfn2 and Drp1 in abdominal aortas (*n* = 6–7). Data were shown as mean ± SD. (one-way ANOVA and the Scheffe post-test. ^*^P < 0.05 versus WT, ^#^*P* < 0.05 versus CKD group).

The mice model of CKD was established by 5/6 nephrectomy. Compared with the sham mice, the CKD mice showed higher level in systolic blood pressure and serum creatinine (Fig. 2A, B). We further stained the kidney sections with PAS stain (Fig. 2C) which showed increased mesangial matrix protein deposition, mesangial cell proliferation, renal tubular atrophy and numerous protein casts in the CKD mice. Meanwhile, the abdominal aortas were harvested and real-time PCR showed that the expression of ANRIL in abdominal aortas was increased in CKD group (Fig. 2D), while BDNF level was significantly decreased (Fig. 2E, G). Fluorescence in situ hybridization (FISH) and CD31 immunofluorescence were used to detect the expression and distribution of ANRIL in aortas (Fig. 2F), which showed that ANRIL was expressed in vascular endothelial cells, and the expression of ANRIL was significantly increased in CKD mice. The endothelial dysfunction was manifested by the induction of proinflammatory cytokines and prothrombotic mediators, like vascular cell adhesion molecule-1 (VCAM-1) and von Willebrand Factor (vWF), along with suppression of endothelial nitric oxide synthase (eNOS) [7]. In this study, compared with aorta from sham mice, aorta from CKD mice exhibited reduced eNOS expression and elevated VCAM-1 and vWF levels (Fig. 2H–J). In addition, mitochondria play a critical role in endothelial function. Normally, mitochondria undergo continuous fission and fusion mainly mediated by dynamin-related protein 1 (Drp1) and mitofusin1/2 (Mfn1/2), respectively. Disruptions in the fission/fusion balance (primarily a shift toward fission) perturb cellular physiology and have been implicated in cardiovascular disease. To determine whether mitochondrial fission is involved in endothelial dysfunction in vivo, we explored the expression of Drp1 and Mfn2 in endothelium via real-time PCR. Aortas from CKD mice exhibited increased Drp1 expression and decreased levels of Mfn2 (Fig. 2K).

To examine the role of ANRIL, we used ANRIL^−/−^ mice to establish CKD model. Although ANRIL^-/-^ mice were phenotypically normal and had no appreciable defect in renal morphology and function, ANRIL knockout ameliorated renal injury as evidenced by the decreased levels of serum creatinine and systolic pressure (Fig. 2A, B). Further experiment confirmed that the knockout of ANRIL accomplished increased expression of eNOS and decreased expression of VCAM-1 and vWF (Fig. 2H–J). Additionally, ANRIL^-/-^ increased the expression of BDNF (Fig. 2E, G). Meanwhile, ANRIL deficiency reduced mitochondrial fission through the Drp1and Mfn2 levels recovered (Fig. 2K). These results clarified that inhibition of ANRIL alleviates endothelial dysfunction in CKD mice models.

### CKD serum induced endothelial dysfunction and ANRIL expression in vitro

**Fig. 3:**
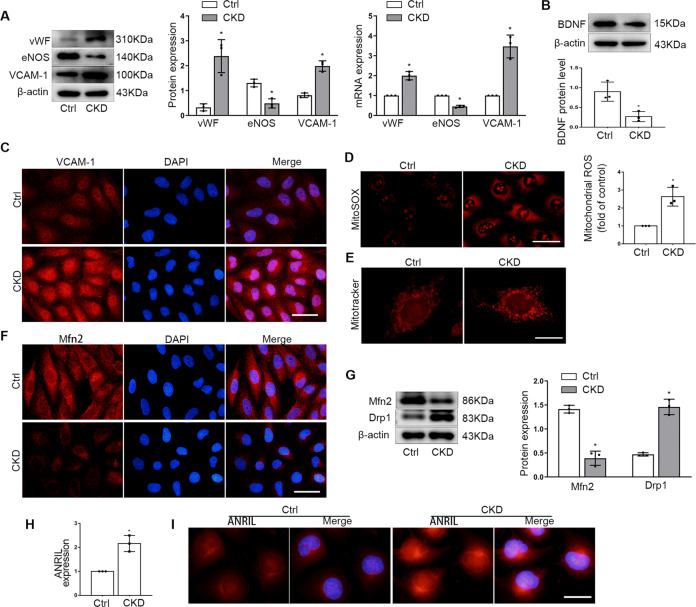
CKD serum induced endothelial dysfunction and ANRIL upregulation. **A** Protein and mRNA levels of vWF, eNOS, VCAM-1 in HUVECs exposed to CKD serum. **B** Protein levels of BDNF in HUVECs exposed to CKD serum. **C** Representative immunofluorescence images of VCAM-1 in HUVECs exposed to CKD serum. Scale bars = 50 μm. **D** Mitosox Red staining showed the mitochondrial ROS level of endothelial cells. Scale bars = 50 μm. **E** Mitotracker Red probe was used to label mitochondria. Scale bars = 20 μm. **F** Representative immunofluorescence images of Mfn2 in HUVECs exposed to CKD serum. Scale bars = 50μm. **G** Expression of Mfn2, Drp1 in HUVECs were detected by Western blot. **H** ANRIL expression in HUVECs were detected by realtime PCR. **I** FISH assays was used to detect the expression and subcellular location of ANRIL in endothelial cells. Scale bars = 25 μm. Data were shown as mean ± SD (*n* = 3). (Student’s *t* test, ^*^P < 0.05 versus control).

Next, to establish an in vitro model of CKD, we examined the effect of uremic serum on endothelial cells by incubating cultured human umbilical vein endothelial cells (HUVECs) with sera from patients in CKD. The proinflammatory and prothrombotic effects of endothelial cells were examined, the results showed that compared with the control serum treatment, the expression of eNOS decreased and the expression of VCAM-1 and vWF increased (Fig. 3A, C). Besides, it was found that CKD serum reduced BDNF expression (Fig. 3B).

Many studies have demonstrated that reactive oxygen species (ROS) derived from mitochondria plays an active role in endothelial dysfunction, and mitochondrial fission is an upstream causal factor for ROS overproduction [21]. Mitosox Red was used to detect mitochondrial ROS production in endothelial cells, and it was found that mitochondrial ROS level was significantly increased in the CKD serum treated group (Fig. 3D). Then Mitochondrial staining was performed with Mitotracker Red fluorescence dye, the result indicated that normal mitochondria were elongated and tubular in shape forming highly interconnected networks, whereas CKD serum induced numerous round mitochondrial fragments (Fig. 3E). Further, the expression of key protein related to mitochondrial fission were detected by Western blot and immunofluorescence staining. The results showed that Drp1 expression increased and Mfn2 expression decreased (Fig. 3F, G), suggesting that CKD serum stimulation can cause mitochondrial fission abnormalities in endothelial cells.

Then, ANRIL expression was detected, and the real-time PCR results showed that ANRIL expression was significantly increased (Fig. 3H). FISH staining was further performed to detect the expression and distribution of ANRIL in cells, which showed that ANRIL was distributed in both the cytoplasm and nucleus (Fig. 3I), suggesting that CKD serum or factors in CKD serum solution could induce endothelial injury and high expression of ANRIL.

### Uremia toxin induced endothelial dysfunction and ANRIL expression in vitro

**Fig. 4:**
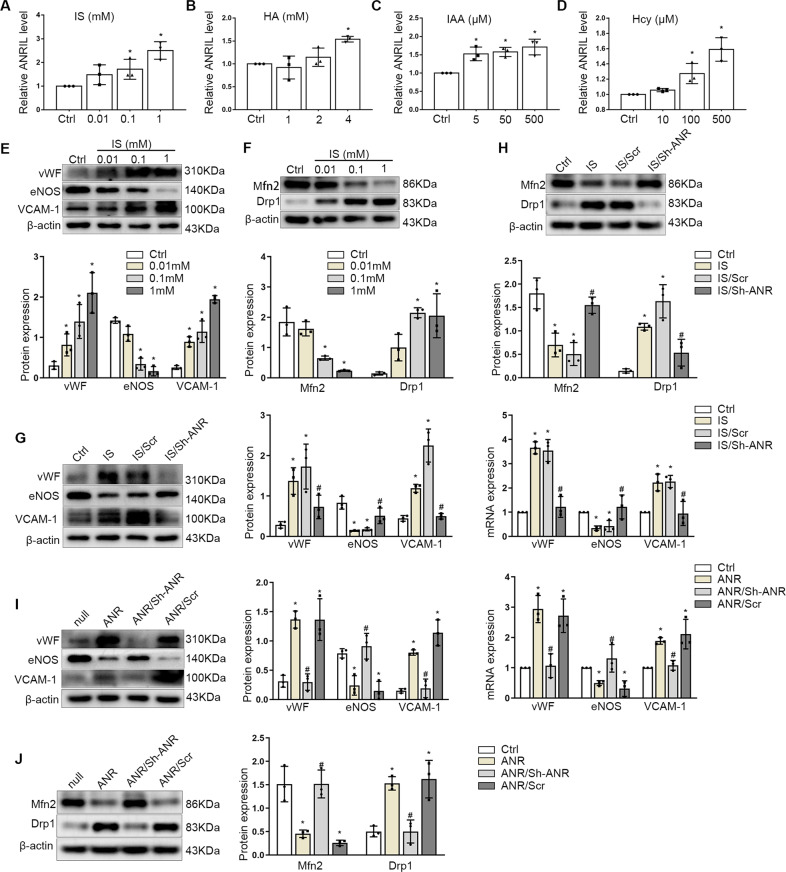
Uremia toxin induced ANRIL expression and ANRIL mediated endothelial dysfunction. **A**–**D** ANRIL expression in endothelial cells stimulated with (**A**) IS, (**B**) HA, (**C**) IAA, (**D**) Hcy at the concentrations indicated for 48 h. **E** Protein levels of vWF, eNOS, VCAM-1 in HUVECs exposed to IS at different doses. **F** Protein levels of Mfn2, Drp1 in HUVECs exposed to IS at different doses. **G** Protein and mRNA levels of vWF, eNOS, VCAM-1 in HUVECs infected with Sh-ANRIL ShRNA under the IS stimulation. **H** Protein levels of Mfn2, Drp1 in HUVECs. **I** Protein and mRNA levels of vWF, eNOS, VCAM-1 in HUVECs with ANRIL overexpression or inhibition. **J** Expression of Mfn2, Drp1 in HUVECs were detected by Western blot. Data were shown as mean ± SD (*n* = 3). (one-way ANOVA and the Tukey post-test. ^*^P < 0.05 versus respective control, ^#^*P* < 0.05 versus IS or ANRIL overexpression). Sh-ANR: Sh-ANRIL infection, Scr: Scramble, ANR: ANRIL overexpression.

Uremic toxin accumulation plays an important role in endothelial dysfunction in CKD, the protein-bound uremic toxins, such as indoxyl sulfate (IS), hippuric acid (HA), indole-3-acetic acid (IAA) and Homocysteine(Hcy) can cause vascular endothelium damage. We incubated ECs with IS, HA, IAA and Hcy. As shown in Fig. 4A–D, uremic toxins could increase the expression of ANRIL dose-dependently. As an important toxin linked to endothelial dysfunction in CKD and increased ANRIL expression significantly, we selected IS for subsequent experiments. To verify the role of IS in endothelial dysfunction, we stimulated endothelial cells with different concentrations of IS. The results showed that IS stimulation could decrease the expression of eNOS, and increase the expression of VCAM-1 and vWF in a concentration-dependent manner (Fig. 4E). In addition, the changes of proteins related to mitochondrial dynamics were detected, and it was found that IS could increase the expression of Drp1 and decrease the expression of Mfn2 (Fig. 4F). The results indicate that uremic toxin in CKD serum induced endothelial dysfunction and ANRIL upregulation.

### ANRIL mediated endothelial dysfunction through BDNF in vitro

To further explore the mechanisms by which uremic toxins induce endothelial dysfunction, we investigated the effects of ANRIL in endothelial injury. We knockdown the expression of ANRIL in endothelial cells under IS stimulation, and the results showed that compared with scramble, ANRIL shRNA lentivirus infection (Sh-ANRIL) lead to eNOS expression upregulated, while the expression of VCAM-1 and vWF decreased (Fig. 4G). In addition, the inhibition of ANRIL also reversed the increase in Drp1 expression and up-regulate Mfn2 expression (Fig. 4H), suggesting that ANRIL could mediate endothelial dysfunction caused by IS stimulation.

To further verify this inference, we infected the endothelial cells with ANRIL overexpression lentivirus (ANRIL), and Sh-ANRIL co-infection reversed its high expression. The results showed that compared with ANRIL overexpression, the Sh-ANRIL double infection increased the expression of eNOS, decreased the expression of VCAM-1 and vWF (Fig. 4I). Correspondingly, knocking down ANRIL can reverse the abnormal expression of Drp1 and Mfn2 caused by ANRIL overexpression (Fig. 4J). These further suggested that ANRIL participated in endothelial dysfunction.

**Fig. 5:**
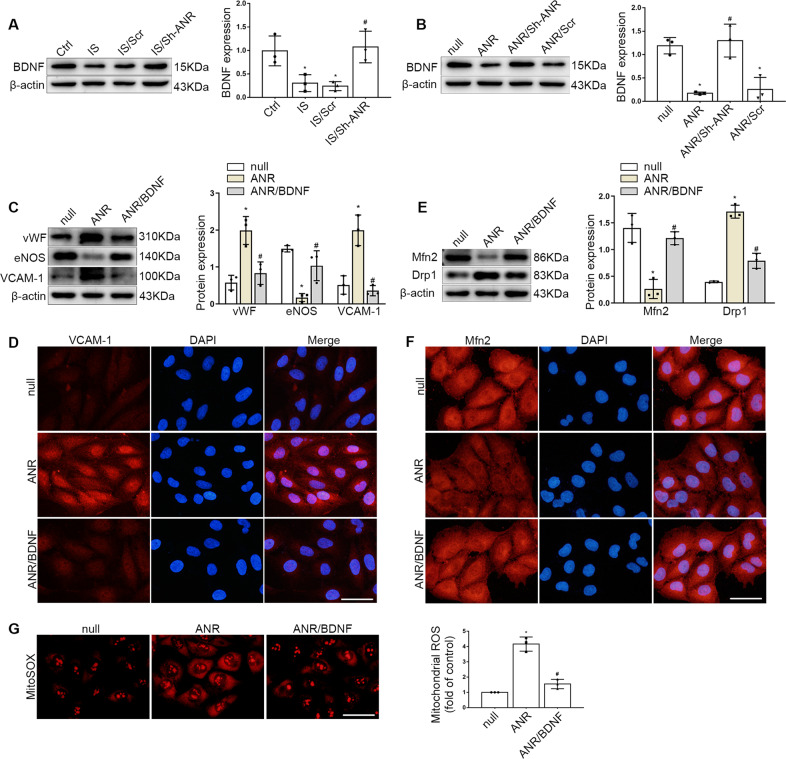
ANRIL mediated endothelial dysfunction through BDNF downregulation. **A** The expression of BDNF in HUVECs infected with ANRIL shRNA under the IS stimulation. **B** Protein levels of BDNF in HUVECs with ANRIL overexpression or inhibition. **C** Protein levels of vWF, eNOS, VCAM-1 in HUVECs. **D** The expression of VCAM-1 was detected by immunofluorescence assay. Scale bars = 50 μm. **E** Protein levels of Mfn2, Drp1 in HUVECs. **F** The expression of Mfn2 was detected by immunofluorescence assay. Scale bars = 50 μm. **G** Mitosox Red staining showed the mitochondrial ROS level of endothelial cells. Scale bars = 50 μm. Data were shown as mean ± SD (*n* = 3). (one-way ANOVA and the Tukey post-test. ^*^P < 0.05 versus respective control, ^#^*P* < 0.05 versus IS or ANRIL overexpression). ANR: ANRIL overexpression, BDNF: BDNF overexpression.

We next analyzed the BDNF level in endothelial cells after ANRIL regulation, the results showed that Sh-ANRIL infection reversed BDNF abnormal expression induced by IS stimulation or ANRIL overexpression (Fig. 5A, B). Then the endothelial cells in ANRIL overexpressed group were transiently transfected with BDNF plasmid, as shown in the results, compared with ANRIL overexpression alone, BDNF upregulation could dramatically reverse the abnormal expression of endothelial and mitochondrial key proteins described above (Fig. 5C–F). Besides, the mitochondrial ROS production was also reduced in BDNF plasmid co-transfected cells (Fig. 5G). Thus, it indicated that ANRIL mediated endothelial dysfunction through BDNF downregulation.

### ANRIL regulated BDNF expression by recruiting EZH2 to the promoter region

**Fig. 6:**
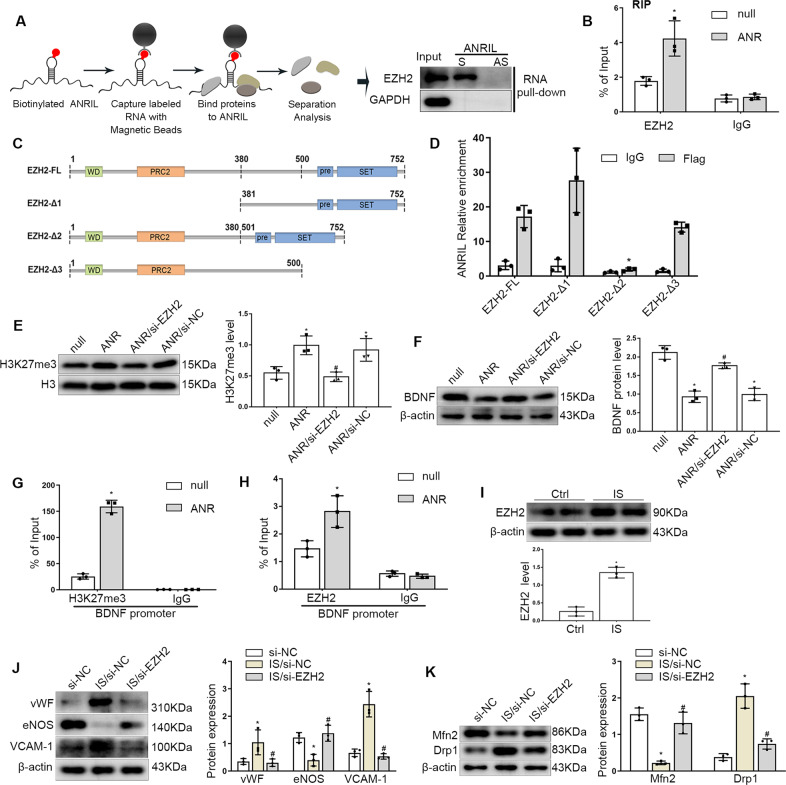
ANRIL regulated BDNF expression by recruiting EZH2 to the promoter region. **A** EZH2 was pulled down by biotin-labeled sense ANRIL (S) but not ANRIL antisense (AS) RNA in HUVECs. **B** RIP assays were applied using anti-EZH2 antibodies with extractions from HUVECs. Relative enrichment represents the RNA levels associated with the indicated protein relative to an input control from three independent experiments after immunoprecipitation with the anti-EZH2 antibody or IgG antibody. **C** Schematic structures of EZH2 proteins and three truncated mutants of EZH2 used in this study. **D** HUVECs transfected with vectors expressing the Flag-tagged FL and the truncated mutants (Δ1−Δ3) of EZH2, then RIP assays were performed using anti-FLAG antibodies. **E** Western blot was used to detect the H3K27me3 level in endothelial cells under the regulation of ANRIL and EZH2. **F** The BDNF level in endothelial cells. **G** ChIP assays showed the H3K27me3 levels at the BDNF promoter region. **H** ChIP assays showed the EZH2 levels at the BDNF promoter region. **I** The expression of EZH2 in HUVECs exposed to IS. **J** Protein levels of vWF, eNOS, VCAM-1 in HUVECs with EZH2 inhibition under IS stimulation. **K** Protein levels of Mfn2, Drp1 in HUVECs. Data were shown as mean ± SD (*n* = 3). (Student’s *t* test, one-way ANOVA and the Tukey post-test. ^*^P < 0.05 versus respective control, ^#^*P* < 0.05 versus ANRIL overexpression or IS/si-NC). ANR: ANRIL overexpression.

Studies have found that ANRIL could act as a recruiter of polycomb repressive complex (PRC) complexes to facilitate altering of chromatin structure and participate in epigenetic transcriptional repression [22, 23]. Enhancer of zeste homolog 2 (EZH2), the main catalytic subunit of PRC2, induces trimethylation of lysine 27 on histone H3 (H3K27me3), represses gene transcription, has been confirmed to affect endothelial cell functions. To study whether ANRIL regulates target genes through this mechanism, we focused on the relationship between ANRIL and EZH2. RNA pull-down combined with western blot was used to detect the ANRIL binding protein, the results showed that EZH2 was pulled down by biotin-labeled ANRIL sense, while the ANRIL antisense sequence does not (Fig. 6A). This effect indicated that ANRIL could directly bind to EZH2. Meanwhile, RNA-binding protein immunoprecipitation (RIP) assays were performed using anti-EZH2 antibodies, which further verified the bound of RNA with EZH2, moreover, the overexpression of ANRIL accentuated this effect (Fig. 6B). To identify the regions of EZH2 responsible for its binding with ANRIL, according to the CatRAPID database, we established three FLAG-tagged vectors containing fragments of EZH2 (Fig. 6C), the RIP assay demonstrated that ANRIL binds the 381–500 amino acid region of EZH2 (Fig. 6D).

Further we found that ANRIL overexpression could increase H3K27me3 level, while this could be reversed by transfection of EZH2 siRNA (Fig. 6E), which suggested that ANRIL could regulate histone modification through EZH2. Meanwhile, we also found that EZH2 inhibition could alleviate the low level of BDNF induced by ANRIL overexpression (Fig. 6F). Remarkably, we found that H3K27me3 levels increased at the BDNF promoter region (Fig. 6G) and the EZH2 levels also increased when ANRIL expression upregulated (Fig. 6H), suggesting that ANRIL regulates BDNF expression by recruiting EZH2 to the promoter region.

The expression of EZH2 in endothelial cells was also increased after uremia toxin stimulation (Fig. 6I). To verify its role in endothelial dysfunction, endothelial cells were transiently transfected with EZH2 siRNA (si-EZH2), or negative control (si-NC), the results showed that compared with si-NC, si-EZH2 increased the expression of eNOS and reversed the upregulation of VCAM-1 and vWF induced by IS stimulation (Fig. 6J). Also, the Drp1 expression was decreased and Mfn2 expression was upregulated after the EZH2 expression inhibited (Fig. 6K). These results suggested that EZH2 could mediate mitochondrial injury and endothelial dysfunction induced by uremia toxin stimulation.

### ANRIL regulated endothelial dysfunction by recruiting EZH2 to the promoter region of BDNF

**Fig. 7:**
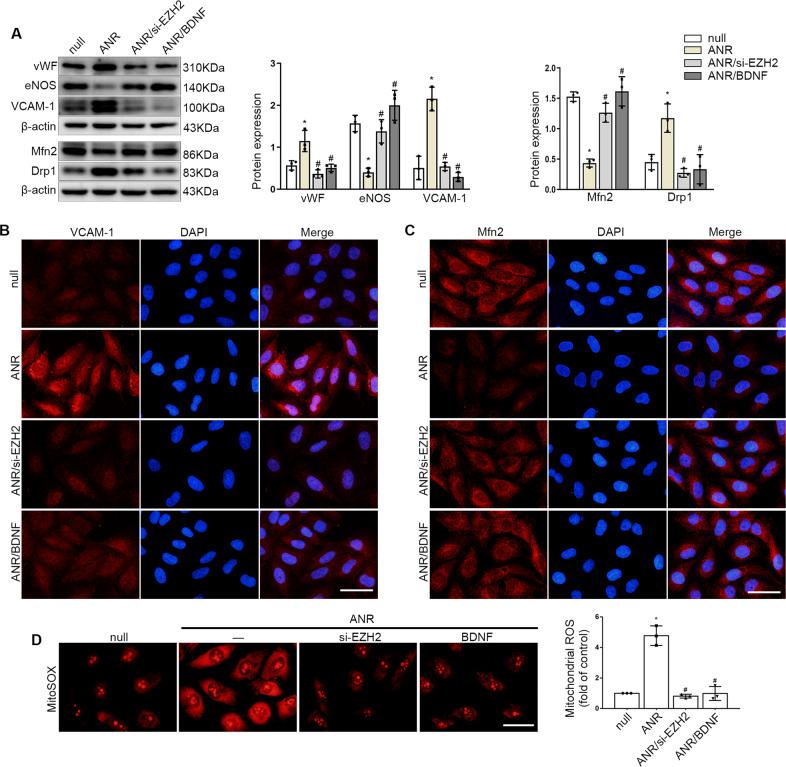
ANRIL mediated endothelial dysfunction by recruiting EZH2 to the promoter region of BDNF. **A** The protein expression of vWF, eNOS, VCAM-1, Mfn2 and Drp1 in HUVECs. **B**, **C** The expression of VCAM-1 and Mfn2 was detected by immunofluorescence assay. Scale bars = 50 μm. **D** Mitosox Red staining showed the ROS level in mitochondria of endothelial cells. Scale bars = 50μm. Data were shown as mean ± SD (*n* = 3). (one-way ANOVA and the Tukey post-test. ^*^P < 0.05 versus respective control, ^#^*P* < 0.05 versus ANRIL overexpression). ANR: ANRIL overexpression, BDNF: BDNF overexpression.

Finally, we verified the role of ANRIL in endothelial dysfunction. The results showed that EZH2 inhibition or BDNF upregulation could reverse the low expression of eNOS and Mfn2 caused by ANRIL overexpression, and the expression levels of vWF, VCAM-1 and Drp1 could be reduced (Fig. 7A–C). Meanwhile, compared with the ANRIL overexpression group, the level of mitochondrial ROS decreased after EZH2 inhibition or BDNF upregulation (Fig. 7D). All these data demonstrated that ANRIL plays an important role in mitochondrial injury and endothelial dysfunction through recruiting EZH2 to the promoter region of BDNF.

## Discussion

The incidence of cardiovascular disease in patients with CKD significantly increased [24], and patients with advanced CKD have more severe vascular damage than those with hypertension or coronary artery disease alone [25]. Endothelial dysfunction which is widespread in cardiovascular diseases and associated with poor prognosis has attracted more attention. Clinically, vascular endothelial function usually refers to its vasodilation ability, especially vasodilation in response to NO, which diffuses into endothelial cells and leads to increased production of cyclic guanosine monophosphate and vasodilation [26]. FMD is currently the most commonly used clinical method for assessing endothelial function, and impaired FMD has been observed in patients with CKD. As NO is produced by eNOS, a unique isoform of NO synthase, the decreased eNOS level is generally regarded as a marker of impaired endothelial function. In addition to the impaired vasodilator capacity, endothelial dysfunction also involves disturbances in antithrombotic, profibrinolytic, and anti-inflammatory and antioxidant properties of the normal endothelium [27]. Therefore, markers of endothelial proinflammatory activation including the cell surface expression of adhesion molecules like VCAM-1 and prothrombotic mediators like vWF [7] are important signs of endothelial dysfunction. In this study, we detected the expression of biomarkers in vascular endothelial cells which confirmed that vascular endothelial dysfunction existed in CKD, and the investigation of endothelial dysfunction in CKD can provide more evidence for the cardiovascular complications in this high-risk population.

Mitochondria, the center of cell energy metabolism, has been proved to be involved in regulating cell apoptosis, calcium processing, innate immunity and phospholipid synthesis. Mitochondria is a dynamic organelle whose dynamic characteristics include fusion, fission and degradation. The interplay of fusion and fission confers widespread benefits on mitochondria, including efficient transport, increased homogenization of the mitochondrial population and efficient oxidative phosphorylation, while the disruption of mitochondrial dynamics and mitochondrial fragmentation results in mitochondrial dysfunction [28]. Accumulating evidence suggests that mitochondrial damage and dysfunction plays a causative role in the development of endothelial dysfunction [29]. Mitochondria are the main source of cellular ROS [30]. The intact mitochondrial membrane structure helps prevent excessive ROS production, while when mitochondrial division is dominant, ROS production increased. Overloaded ROS can promote cell inflammation, proliferation and apoptosis through a variety of signaling pathways [31]. A variety of proteins are involved in the regulation of mitochondrial dynamics. The mitofusins, Mfn1 and Mfn2, are located on the mitochondrial outer membrane and are required for outer membrane fusion, while the inner membrane fusion is mediated by Optic Atrophy 1. The central mediator of mitochondrial fission is Drp1, a GTP-hydrolyzing enzyme, which can be recruited from a cytosolic pool onto the mitochondrial surface, where it self-assembles into spiral structures that wrap around and constrict mitochondrial tubules to facilitate fission [32, 33]. In the current study, the elevated mitochondrial ROS levels and increased Drp1 protein expression were observed in endothelial cells exposed to CKD serum and CKD mice, which suggested that CKD could induce mitochondrial injury. Studies have shown that ROS-mediated oxidative stress induced eNOS degradation and NO production decrease [29]. Drp1 and its mitochondrial Receptor Mff mediate inflammatory NF-κB activation and VCAM-1 induction in endothelial cells [34]. Therefore, mitochondrial injury was associated with the progression of endothelial dysfunction and the mechanism need to be further researched.

Chronic inflammation and oxidative stress are key mechanisms of endothelial dysfunction in patients with CKD. The uremic toxins accumulated in CKD have been identified as inducers of inflammation, oxidative stress and endothelial dysfunction. Protein-bound uremia toxins can damage vascular endothelial cells due to poor clearance by dialysis, and induce the transform into a pro-oxidative and proinflammatory phenotype [35]. To identify the components that induced the increase of ANRIL in CKD serum, we then incubated endothelial cells with IS, HA, IAA and Hcy, and found that uremic toxins could increase the expression of ANRIL dose-dependently, which suggested that the upregulation of ANRIL in CKD was due to or at least partly due to uremia toxin. IS has been proved to cause increased oxidative stress, inhibit NO production, induce endothelial inflammation and thrombosis. Also, we showed that IS stimulation induced abnormal expression of proteins related to endothelial injury and mitochondrial fission in a concentration-dependent manner, and IS was selected for mechanism experiments.

Accumulating studies indicate that lncRNAs play important roles in the pathobiology of cardiovascular diseases [36]. Further studies have shown that lncRNAs can induce cardiovascular disease by mediating endothelial dysfunction. For instance, MALAT1 was upregulated in patients with atherosclerosis, while its suppression protected the endothelium from oxLDL-induced inflammation and oxidative stress by upregulation of miR-181b and downregulation of thymocyte selection-associated high mobility group box [37]. HOTAIR served as a scaffold for histone modification complexes PRC-2 and LDS-1, facilitating epigenetic histone modifications, and its overexpression could reduce endothelial dysfunction in atherosclerosis [38]. Research found that there were multiple subtypes of ANRIL, and the linear ANRIL subtype was mainly expressed in patients carrying the risk allele of coronary heart disease. Moreover, ANRIL expression in plaque, monocytes or whole blood correlated with atherosclerosis severity [39]. Abnormal expression of ANRIL could mediate atherosclerotic endothelial injury by combining YY1 or upregulating CARD8 and VEGF [40]. In addition, lncRNAs are stable in plasma and other body fluids and can be used as biomarkers for disease diagnosis [41]. Therefore, in this study, the ANRIL level of clinical plasma samples was measured to evaluate its role in endothelial injury of CKD, and we found that ANRIL was increased in the plasma of CKD patients and negatively correlated with FMD, suggesting that ANRIL was associated with endothelial dysfunction in CKD. Meanwhile, the high expression also could be detected in vitro and in vivo, and ANRIL knockout could alleviate vascular endothelial cell injury, these finding suggested that ANRIL was a key regulator of endothelial injury in CKD. Besides, studies have indicated the role for ANRIL in acute injury. For example, ANRIL expression levels was increased in heart tissues of acute myocardial infarction mice, and regulated myocardial cell apoptosis through IL-33/ST2 pathway [42]. Moreover, research has shown that knockdown of ANRIL can inhibit kidney inflammation in LPS-induced acute kidney injury mice [43]. Therefore, we hypothesized that the role of ANRIL was not limited to chronic stage.

BDNF is a neurotrophin widely expressed in nervous tissue, with a role in nerve growth regulation and synaptic plasticity [44]. Now research confirms that BDNF circulates systemically, and the functional pleiotropy extends beyond the nervous system. BDNF is also a key regulatory factor of cardiovascular disease. Studies have found that patients with coronary artery disease exhibited significantly lower plasma BDNF [14], and the serum BDNF levels were associated with an increased risk of adverse cardiovascular events and death [13, 16]. BDNF is highly expressed in endothelial cells, which can promote vascular development and endothelium budding and induce angiogenesis, and plays an important role in vascular diseases [45]. BDNF mimetic, 7,8-dihydroxyflavone protects against cardiac ischemic injury by inhibiting the mitochondrial excessive fission and cell death [46]. Consistent with previous studies, in our study, serum BDNF levels were significantly decreased in CKD patients and the serum BDNF levels were correlated with eGFR positively, which suggested that it participated in the progression of CKD-related endothelial dysfunction. Furthermore, the ANRIL level in patients with CKD showed a negative correlation with BDNF concentration, and the significant negative correlation between ANRIL and BDNF was also detected in endothelial cells after ANRIL regulation. Meanwhile, BDNF upregulation could ameliorate endothelial and mitochondrial injury induced by ANRIL overexpression which indicated that ANRIL mediated endothelial dysfunction through BDNF, and these also verified its role in cardiovascular complications.

LncRNAs also have secondary and three-dimentional structures which enable them to have both RNA- and protein-like functions. Current studies have found that lncRNAs play an important role in regulating gene expression at epigenetic, transcriptional and post-transcriptional levels [47, 48]. For instance, lncRNAs regulate histone methylation by interacting with PRC2, thereby regulating gene expression. And lncRNAs can interact with chromatin rearrangement complexes to affect chromatin rearrangement. Also lncRNAs can mediate cell function by binding and regulating transcription factors. In addition, studies have found that lncRNAs can stabilize mRNA by recruiting proteins such as STAU1 to prevent degradation [49]. Recent studies show that ANRIL can promote NLRP3 inflammasome activation by upregulating BRCC3 expression via sponging miR-122-5p [50]. Besides, ANRIL can function as a recruiter of PRC complexes to facilitate altering of chromatin structure and regulate the target gene expression [51]. In the current study, RNA pulldown and RIP verified that ANRIL can directly bind to EZH2, the main catalytic subunit of PRC2. There is a wealth of data on the role of EZH2 in promoting renal fibrosis in acute kidney injury induced by ischemia-reperfusion, folic acid, or unilateral ureteral obstruction. The high expression of EZH2 has also been found in humans with CKD. Blockage of EZH2 with pharmacologic inhibitor 3-deazaneplanocin A or siRNA attenuates progression of renal fibrosis [52, 53]. Studies have showed that EZH2 was demonstrated to be related to the occurrence and development of atherosclerosis [54]. LDL-C induces endothelial EZH2 expression and mitigates KLF2-dependent NO production, resulting in vasoconstriction and the decreased expression of anti-atherogenic factors thrombomodulin and plasminogen activator inhibitor-1 [55]. EZH2 was upregulated in the aorta of hyperhomocysteinemic ApoE^-/-^ mice and contributed to foam cell formation [56]. In addition, studies had shown that EZH2 expression was upregulated in plaque areas compared with adjacent plaque-free areas, also EZH2 expression increased in diseased vessels compared with healthy control carotid artery [57]. Likewise, EZH2 expression was increased with uremic toxin stimulation, while the inhibition of EZH2 could reverse endothelial injury. Thus we speculated that EZH2 was involved in the occurrence and development of vascular disease in CKD. The cellular effects of EZH2 was mediated by modulating H3K27me3 level and the binding of this repressive epigenetic mark to target gene promoters [58]. Therefore, in this study, Chromatin immunoprecipitation (ChIP) assays showed that ANRIL overexpression increased EZH2 level within BDNF promoter and aggravated the repressive H3K27me3 epigenetic mark on BDNF gene promoters, suggesting that ANRIL regulated BDNF expression by recruiting EZH2 to the promoter region.

Furthermore, domain, a part of protein sequence and structure, plays an important role in proteins’ functions and interactions. EZH2 consists of four domains, Polycomb repressive complex 2 tri-helical (PRC2) domain, WD-binding domain, SET domain and preSET domain. To identify the regions of EZH2 responsible for its binding with ANRIL, three FLAG-tagged vectors containing fragments of EZH2 were established. We found that ANRIL binds the 381-500 amino acid region of EZH2 which was between PRC2 and preSET domains. So we speculated that ANRIL may function as a scaffold for EZH2 to help play roles in endothelial function.

**Fig. 8:**
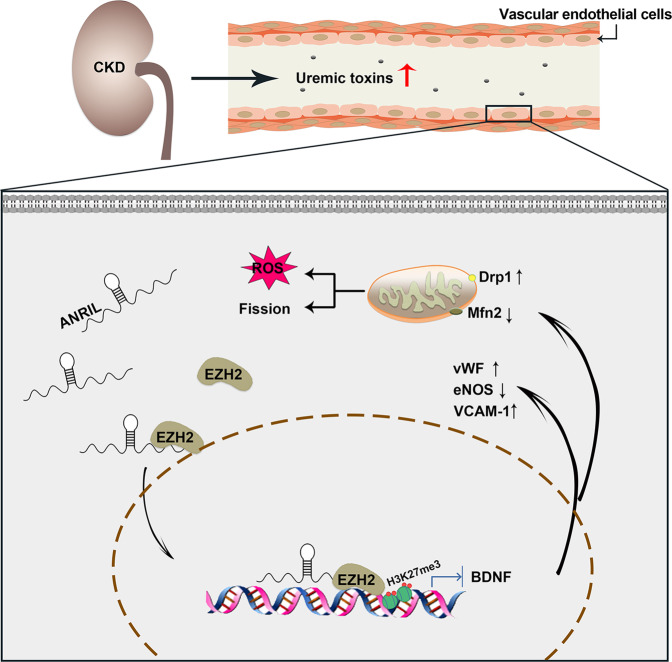
Schematic illustration. Uremic toxin in the serum derived from CKD patients induce overexpression of ANRIL, and ANRIL mediate brain-derived neurotrophic factor (BDNF) promoter methylation through recruitment of EZH2 to the BDNF promoter region, eventually resulting in endothelial dysfunction.

In conclusion, our study found that the circulating ANRIL level was correlated with vascular endothelial dysfunction in patients with CKD. Then our study provides evidence to demonstrate that ANRIL mediated endothelial dysfunction through BDNF downregulation. Our results indicate that ANRIL directly binds to EZH2, and mediates BDNF promoter methylation through recruitment of EZH2 to the BDNF promoter region, eventually resulting in endothelial dysfunction (Fig. 8). These findings provided evidence to suggest that ANRIL was involved in the development of cardiovascular complications in CKD. and the exact molecular mechanisms need further exploration.

## Material and methods

### Patients

Totally, 59 adult patients with various stages of CKD and 45 sex- and age-matched healthy adults were enrolled in this study. Patients were diagnosed as CKD according to National Kidney Foundation K/DOQI Guideline. For the purpose of research, patients with diabetes mellitus, hyperlipidemia, and NYHA heart failure with grade III or IV were excluded, and exclusion criteria also include: failure to provide informed consent, participating in an interventional clinical trial, pregnant or lactating women, patients with acute kidney injury, only simple hematuria, cirrhosis of the liver, HIV infection or AIDS, previous organ or bone marrow transplant, receive chemotherapy or alkylating agent treatment in the past 2 years, received immunosuppressive therapy within the previous 6 months, previous dialysis treatment for more than one month, Nephritis caused by systemic autoimmune diseases was excluded, genetic kidney diseases such as polycystic kidney disease were excluded. The study was approved by Biomedical Research Ethics Committee of Shandong Provincial Hospital affiliated to Shandong University, and all participants provided informed consent.

FMD of right brachial artery was performed as described previously [59] and all examinations were performed by the same skilled technician who was blinded to the patient’s data.

Blood samples were obtained from patients with CKD or control participants by venipuncture. Blood samples were centrifuged at 3000 rpm for 15 min, then plasm were collected for analyses of ANRIL and BDNF levels, and serum were collected for analyses of serum creatinine and cell experiments.

### Animals

Male SPF C57BL/6 mice were obtained from the Experimental Animal Center of Shandong University. The ANRIL knockout (ANRIL^-/-^) C57BL/6 mice model was constructed using CRISPR/Cas9 gene knockout technology (Cyagen Biosciences, Guangzhou, China). The mice were housed in temperature-controlled cages under a 12-h light/12-h dark cycle with free access to food and water. A total of 16 male C57BL/6 mice and 16 male ANRIL^-/-^mice (8 weeks old) enrolled in this study and randomly allocated into the following groups (*n* = 8): Sham, CKD, ANRIL^-/-^ and CKD/ANRIL^-/-^. CKD model was established by 5/6 nephrectomy with a 2-step surgical procedure. Briefly, the upper and lower poles of the right kidney were resected. One week later, the left kidney was removed after ligation of the renal blood vessels and the ureter. For the sham and ANRIL^-/-^ mice, only the adipose capsule was isolated, and the kidney tissue was retained. Blood pressure and weight were monitored, and at the end of 8 weeks, mice were sacrificed, blood, abdominal aorta and kidneys were harvested for following experiments. All animal studies were carried out with the review and approval of the Experimental Animal Ethics Committee of Shandong Provincial Hospital affiliated to Shandong University.

### Cell Culture

HUVECs were originally obtained from Sciencell Research Laboratories, and cultured in endothelial cell medium (Sciencell, California, USA) containing 5% fetal bovine serum, 1% penicillin-streptomycin solution and endothelial cell growth supplement in a 5% CO_2_ incubator. HUVECs at passages 5–8 were used for the in vitro studies. To examined the effect of uremic serum on endothelial cells, sera from patients with CKD or controls were pooled, and diluted to 20% by using ECM culture media, then endothelial cells were incubated for 48 h. IS and Hcy (Sigma-Aldrich, St. Louis, MO), IAA and HA (Solarbio, Beijing, China) were diluted in ECM media and used to treated HUVECs. The cells were verified by short tandem repeat profiling and tested negative for mycoplasma contamination.

### Cell transfection

LncRNA ANRIL lentivirus were constructed from Cyagen Biosciences, include: the overexpression vector (pLV[ANRIL]-EGFP: T2A: Puro-EF1A), control vector (pLV[Exp]-EGFP: T2A: Puro-Null), Scramble shRNA and ANRIL shRNA. The lentivirus infection was performed according to standard protocols. Satisfactory overexpression or knockdown efficiency of ANRIL was confirmed by real-time PCR (Supplementary fig. A, B). The target sequences of shRNA were designed as following:

Scramble shRNA (Scramble): 5′-CCTAAGGTTAAGTCGCCCTCG-3′;

ANRIL shRNA (Sh-ANRIL): 5′-CTCCGCTCCTCTTCTAGATTT-3′.

The full-length expression plasmids of BDNF (pcDNA3.1-BDNF), EZH2 siRNA (si-EZH2) and their negative control inhibitors(si-NC) were purchased from Genomeditech (Shanghai, China). Cell transfection was performed with the Lipofectamine 3000 Transfection Reagent (Invitrogen, USA) following the handbook of transfection reagent. Satisfactory overexpression efficiency of BDNF and knockdown efficiency of EZH2 was confirmed by western bolt (Supplementary fig.). The sequences used for EZH2 inhibition are as follows:

Si-EZH2: 5′-GAGGGAAAGUGUAUGAUAA-3′

5′-UUAUCAUACACUUUCCCUC-3′

siRNA NC: 5′-UUCUCCGAACGUGUCACGU-3′

5′-ACGUGACACGUUCGGAGAA-3′

The full-length expression plasmids of EZH2 (EZH2-FL) and three truncated mutants (Fig. 6C, Δ1: [381-752]; Δ2: [1-380] + [501-752]; Δ3: [1-500]) were constructed from Ribobio Biosciences, cell transfection was performed with the Lipofectamine 3000 Transfection Reagent following the handbook of transfection reagent.

### Histology, immunohistochemistry and immunofluorescence

Renal and abdominal aortic tissues were fixed with 4% paraformaldehyde and the paraffin-embedded sections were used for staining. Renal histological analysis was performed with periodic Acid-Schiff (PAS, Solarbio, China). For immunohistochemical analysis, abdominal aortic sections were incubated with primary antibodies against VCAM-1 (Abcam, ab 134047, 1:500) and stained with 3, 3-diaminobenzidine tetrahydrochloride and hematoxylin reagent (ZSGB BIO, China). For immunofluorescence of abdominal aortic sections, the tissues were incubated with primary antibodies against eNOS (Cell Signaling Technology, 32027, 1:100) and BDNF (Abcam, ab108319, 1:200), followed by co-incubated with Alexa Fluor®594-conjugated Goat Anti-Rabbit IgG (Abcam, ab150076, 1:200) and FITC Anti-Mouse CD31 (Proteintech, FITC-65058, 1:100) antibodies at 37 °C for 90 min the next day. For immunofluorescence of cell, after treatment, fixed cells were immunostained with primary antibodies against VCAM-1 and Mfn2 (Proteintech, 12186-1-AP, 1:200) at 4 °C overnight and Alexa Fluor®594-conjugated Goat Anti-Rabbit IgG the next day. The images were observed with microscope (Olympus, Tokyo, Japan).

### Enzyme-linked immunosorbent assay

Human plasma BDNF concentrations were measured with an enzyme-linked immunosorbent assay kit (Elabscience, China) according to the manufacturer’s recommended instructions. Briefly, Samples (or Standards) were added to the micro plate wells and combined with the specific antibody. Then a biotinylated detection antibody specific for Human BDNF and Avidin-Horseradish Peroxidase conjugate were added successively to each micro plate well and incubated. Then washed and added the substrate solution to each well. The enzyme-substrate reaction was terminated by the addition of stop solution. The optical density was measured at a wavelength of 450 nm. The concentrations of BDNF was calculated from the standard curves. The detection sensitivity of BDNF was 18.75 pg/ml.

### Real-time PCR

Total RNA was extracted from cultured cells or abdominal aortic tissues using TRIzol Reagent (Takara Biotechnology, Japan). The total RNA (1.0 μg each) from each sample were reverse transcribed into cDNA by using a PrimeScript™ RT Reagent Kit (Takara Biotechnology). Then, real-time PCR analysis was performed with SYBR Premix Ex TaqTM (Takara Biotechnology) in the LightCycler ® 480 Real-Time PCR system (Roche Diagnostics, Basel, Switzerland). Specificity was determined by melting-curve analysis, and β-actin was used as an internal reference.

For plasm RNA detection, RNA was extracted from plasm using Plasma/serum free RNA extraction kit (BIOG Biotechnology, China), and reverse transcribed into cDNA, then real-time PCR analysis was performed. The primers used in our study were listed in Supplementary Table 2.

### Western blot

The total protein was extracted from cells under different conditions with RIPA buffer (Beyotime Biotechnology, China) containing proteinase inhibitors and phosphatase inhibitors (Solarbio, China). Protein extracts were separated by SDS-PAGE and transferred to PVDF membranes (Millipore, USA), the membranes were then blocked with 5% milk or BSA for 1 hour and incubated with primary antibodies at 4 °C overnight followed by incubation with horseradish peroxidase-conjugated second antibodies, respectively. Bands were visualized by ECL reagent (Millipore), and imaged by the Amersham Imager 680 (GE, Boston, MA, USA). The integrated optical density of each band was calculated using ImageJ.

The antibodies included: eNOS (D9A5L) Rabbit mAb (Cell Signaling, 32027, 1:1000), Anti-Von Willebrand Factor antibody (Abcam, ab174290, 1:2000), Anti-VCAM1 antibody (Abcam, ab134047, 1:1000), Anti-DRP1 antibody (Abcam, ab184247, 1:1000), Mfn2 Polyclonal antibody (Proteintech, 12186-1-AP, 1:2000), Anti-BDNF antibody (Abcam, ab108319, 1:2000), Anti-KMT6/EZH2 antibody (Abcam, ab228697, 1:1000), Anti-Histone H3 antibody (Abcam, ab1791, 1:1000), Anti-Histone H3 (tri methyl K27) antibody (Abcam, ab192985, 1:1000), β-actin Monoclonal Antibody (Proteintech, 66009-1-Ig, 1:5000), HRP-conjugated Affinipure Goat Anti-Rabbit IgG (Proteintech, SA00001-2, 1:5000), HRP-conjugated Affinipure Goat Anti-Mouse IgG (Proteintech, SA00001-1, 1:5000).

### ChIP

Cells treated with formaldehyde were broken open and sonication was performed to shear the chromatin to a manageable size. Then, add the immunoprecipitating antibody (IgG, EZH2 or H3K27me3) and 20 μL of fully resuspended protein A/G magnetic beads, incubate overnight at 4 °C with rotation. Next, Protein-DNA cross-links were reversed and DNA was purified to remove the chromatin proteins. Immunoprecipitated DNA samples were subjected to quantitative real-time PCR using primers specific to the BDNF promoter. The cumulative fluorescence for each amplicon was taken as a percentage of the input fraction. DNA primers were as follows: BDNF promoter (−753 to −480): Forward-CACAGGGAGATGCAAGTTGA; reverse-GAAAGGCACTCCCATTTCAG.

### RIP

An RIP experiment was performed according to the instructions of the Magna RIP RNA Binding Protein Immunoprecipitation Kit (Millipore, 17-701, USA). Briefly, lysate was prepared in a lysis buffer containing protease inhibitor cocktail and RNase inhibitor. Then, protein A/G magnetic beads were prepared for incubation with 5 μg of purified antibodies per immunoprecipitation with rotation for 30 min at room temperature. Further, to precipitate RNA-binding protein-RNA complexes, the mixture was incubated with rotation overnight at 4 °C. Finally, RNA was purified using proteinase K buffer and subjected to quantitative real-time PCR. The RNA levels were normalized to the input RNA levels.

### RNA pulldown

Expression vectors for full-length ANRIL used for the in vitro synthesis of RNA were provided by GenePharma Technology. The lncRNAs were transcribed in vitro using a MAXIscript™ SP6/T7 Transcription Kit (Invitrogen, AM1320, USA) and were biotinylated with a Pierce RNA 3′ End Desthiobiotinylation Kit (Thermo Fisher Scientific, 20163) according to the manufacturer’s instructions. The proteins were extracted from HUVECs using Pierce IP Lysis Buffer (Thermo Fisher Scientific). Then, RNA pull-down assays were performed with a Pierce Magnetic RNA-Protein Pull-Down Kit (Thermo Fisher Scientific, 20164). Briefly, the biotinylated lncRNAs were captured with streptavidin magnetic beads and incubated with the cell lysates at 4 °C overnight. Then, the mixture was washed and eluted. The eluate was subjected to Western blotting analysis.

### Mitochondrial ROS analysis

The levels of mitochondrial ROS (mitoROS) in HUVECs were detected using MitoSOX Red dye (Yeasen, China). After treatment, cells were incubated in Hank’s balanced salt solution containing 5 μmol/L MitoSOX Red at 37 °C for 10 min in the dark. After incubation, the cells were rinsed twice with PBS to remove the dye. The fluorescence of MitoSOX Red was detected by fluorescence microscopy (Olympus). Quantification of fluorescence intensity was measured using ImageJ software.

### Mitochondrial morphology analysis

The change in mitochondrial morphology was detected using MitoTracker® Red Probe (Cell Signaling, 9082, USA), the probe was soluted according to the instructions. After treatment, the stock solution was directly diluted into growth media at a concentration of 100 nM, then cells were incubated for 30 min at 37 °C. After incubation, fix the cells in ice-cold methanol for 15 min then rinse 3 times with PBS for 5 min. The mitochondrial morphology was detected by Microscopy (Olympus Microsystems, Tokyo, Japan).

### FISH

For cell RNA FISH assay, hsa-ANRIL probe and FISH kit were purchased from Ribio (Guangzhou, China). Briefly, cultured HUVECs under different conditions were plated onto 6-well plates and fixed in 4% paraformaldehyde for 10 min, followed by ice-cold PBS containing 0.5% Triton X-100 for 5 min, and then incubated with pre-hybridization buffer for 30 min at 37 °C to block non-specific binding. Hybridization buffer was preheated in a 37 °C water bath, and the ANRIL FISH probe working buffer was prepared by diluting probe stock solution in hybridization buffer (1:50). Cells were incubated with probe working buffer at 37 °C overnight and followed by DAPI staining to visualize the nuclei. Images were observed and captured on an inverted fluorescence microscope (Olympus Microsystems).

For abdominal aorta assay, mus-ANRIL probe and fluorescence in Situ Hybridization kit were purchased from Ribio. Briefly, the mouse abdominal aortas were paraffin embedded, sectioned at 3μm, and subjected to antigen retrieval followed by blocking with pre-hybridization buffer for 30 min at 37 °C to block non-specific binding, the mus-ANRIL FISH probe working buffer was prepared by diluting probe stock solution in hybridization buffer (1:50), and tissues were incubated with probe working buffer at 37 °C overnight. Next, tissues were washed and blocked with 1% BSA for 30 min at room temperature, then incubated with CD31 rabbit primary pAb (Abcam, ab28364, 1:100) overnight at 4 °C. Alexa Fluor® 488-conjugated goat anti-rabbit IgG (Abcam, ab150077, 1:200) antibody was subsequently used for immunodetection. The images were observed with microscope.

### Statistical analysis

Data were analyzed using SPSS 19.0 software. Categorical variables were compared using the chi-square test. Measurement data were expressed as mean ± SD. Statistical significance between two groups was assessed using Student’s *t* test or the Mann–Whitney *U* test according to the normality evaluation. Statistical comparisons among multiple groups were conducted using one-way ANOVA followed by Tukey post-test for equal sample sizes or the Scheffe post-test for unequal sample sizes. The correlation between measurement data was assessed by Spearman’s rho. A value of *p* < 0.05 was considered statistically significant.

## Supplementary information

Author Contribution Statement

aj-checklist

Table1. Baseline demographic and clinical characteristics

Table2. Primers for real time PCR

Supplementary Figure.

Supplementary Figure Legend

Original western blots

Original western blots

Original western blots

Original western blots

Original western blots

Original western blots

## Data Availability

The datasets used and/or analyzed during the current study are available from the corresponding author on reasonable request.
