# Supplementary material for: LncRNA ANRIL mediates endothelial dysfunction through BDNF downregulation in chronic kidney disease
Source: Cell Death Dis. 2022 Jul 29;13(7):661. doi: 10.1038/s41419-022-05068-1 (PMC9338026; doi:10.1038/s41419-022-05068-1)
Supplement: Supplementary file 1 — Author Contribution Statement [file 41419_2022_5068_MOESM1_ESM.pdf]

DECLARATION OF CONTRIBUTIONS TO ARTICLE

ADMC

Manuscript Number: CDDIS-22-1206RR

Journal Name: *Cell Death & Disease* (the 'Journal')

Proposed Title of the Contribution:

LncRNA ANRIL mediates endothelial dysfunction through BDNF downregulation in chronic kidney disease (the 'Contribution)

Author(s):

Hong Su, Bing Liu, Huimin Chen, Tingwei Zhang, Tongtong Huang, Yue Liu, Cheng Wang, Qiqi Ma, Qianhui Wang, Zhimei Lv, Rong Wang (the 'Authors')

For all *CDDis* articles, each person named as an author in the published version must be able to show he or she has contributed substantially to the article.

Authorship credit should be based on 1) substantial contributions to conception and design, acquisition of data, or analysis and interpretation of data; 2) drafting the article or revising it critically for important intellectual content; and 3) final approval of the version to be published. Authors should meet conditions 1, 2 and 3.

Any person who cannot be shown to have made a substantial contribution to the article cannot be listed as an author in the final version. The name of any person who is deemed to have made a minor contribution can, however, appear in the Acknowledgments section of the article.

Please complete the table below to indicate the contributions of all named authors to the manuscript.

Author Full Name:	Specification of Contribution to the Manuscript:
Hong Su	acquisition of data, analysis and interpretation of data, drafting the article
Bing Liu	acquisition of data, analysis and interpretation of data, drafting the article
Huimin Chen	acquisition of data, analysis and interpretation of data
Tingwei Zhang	acquisition of data, analysis and interpretation of data
Tongtong Huang	acquisition and analysis of data
Yue Liu	acquisition and analysis of data
Cheng Wang	acquisition and analysis of data
Qiqi Ma	acquisition of data
Qianhui Wang	acquisition of data
Zhimei Lv	conception and design, final approval of the version to be published
Rong Wang	conception and design, final approval of the version to be published

Please complete the table below to indicate the contributions of all named authors to the figures.

Figure 1:

ZL and RW designed the study and established the inclusion/exclusion criteria. QM, QW and YL assisted in clinical sample collection and data analysis, HS and BL performed the BDNF and ANRIL detection.

Figure 2:

TZ and CW established the CKD model and collected blood, abdominal aorta and kidneys for following experiments. HS and BL performed development of methodology and histological analysis.

Figure 3:

HS and BL performed cell culture, determination of experssion levels of eNOS, vWF, VCAM-1, Drp1, Mfn2 and ANRIL. HC and TH assisted in mitochondrial ROS and morphology analysis.

Figure 4:

HS and BL performed cell transfection, determination of experssion levels of eNOS, vWF, VCAM-1, Drp1and Mfn2. HC and TH assisted in ANRIL expression analysis in cells treated with uremic toxin (IS, HA, IAA and Hcy).

Figure 5:

HS and BL performed cell transfection, determination of expression levels of eNOS, vWF, VCAM-1, Drp1 and Mfn2. HC and TH assisted in mitochondrial ROS analysis.

Figure 6:

HS and BL performed cell transfection, RNA pull-down, RIP and Chip assays. HC and TH tested the protein levels of eNOS, vWF, VCAM-1, Drp1, Mfn2, EZH2, H3K27me3 and BDNF.

Signed for and on behalf of the Author(s):	Print Name:	Date:
lv zhimei	Zhimei Lv	2022-07-02
