# Supplementary material for: LncRNA ANRIL mediates endothelial dysfunction through BDNF downregulation in chronic kidney disease
Source: Cell Death Dis. 2022 Jul 29;13(7):661. doi: 10.1038/s41419-022-05068-1 (PMC9338026; doi:10.1038/s41419-022-05068-1)
Supplement: Supplementary file 3 — Table1. Baseline demographic and clinical characteristics [file 41419_2022_5068_MOESM3_ESM.docx]

Table1. Baseline demographic and clinical characteristics

|  | CKD, n=59 | Control, n=45 | P |
| --- | --- | --- | --- |
| Age, years | 48.9±15.1 | 46.2±12.4 | 0.326 |
| Men: Women | 41:18 | 34:11 | 0.494 |
| SBP, mmhg | 140.5±17.3 | 124.6±7.6 | ＜0.001* |
| DBP, mmhg | 89.2±11.0 | 84.7±6.5 | 0.025 |
| Serum creatine, μmol/L | 184.8±92.5 | 70.6±14.5 | ＜0.001* |
| eGFR, ml/min/1.73m^2^ | 40.58±19.02 | 101.33±19.91 | ＜0.001* |
| Total cholesterol, mmol/L | 5.12±1.51 | 5.23±0.73 | 0.662 |
| Triglyceride, mmol/L | 2.04±1.49 | 1.57±0.67 | 0.073 |
| HDL-c, mmol/L | 1.19±0.40 | 1.20±0.29 | 0.913 |
| LDL-c, mmol/L | 3.09±0.98 | 3.09±0.58 | 0.950 |
| Fasting glucose, mmol/L | 5.42±0.85 | 5.40±0.70 | 0.931 |
| FMD, % | 5.52±3.79 | 7.79±4.68 | 0.037* |
| BDNF, pg/mL | 813.94±796.50 | 2179.06±1184.10 | ＜0.001* |

Note: SBP: systolic pressure, DBP: diastolic pressure, eGFR: estimated glomerular filtration rate, HDL-c: high density lipoprotein cholesterol, LDL-c: low density lipoprotein cholesterol, FMD: Flow-mediated dilatation, BDNF: brain-derived neurotrophic factor.
