# Supplementary material for: LncRNA ANRIL mediates endothelial dysfunction through BDNF downregulation in chronic kidney disease
Source: Cell Death Dis. 2022 Jul 29;13(7):661. doi: 10.1038/s41419-022-05068-1 (PMC9338026; doi:10.1038/s41419-022-05068-1)
Supplement: Supplementary file 4 — Table2. Primers for real time PCR [file 41419_2022_5068_MOESM4_ESM.docx]

Table 2. Primers for real time PCR

| Gene | Primer sequence |
| --- | --- |
| eNOS | CGAGTGAAGGCGACAATCCT  CGAGGGACACCACGTCATAC |
| vWF | TTGACGGGGAGGTGAATGTG  ATGTCTGCTTCAGGACCACG |
| VCAM1 | GGGAAGCCGATCACAGTCAA  GGGACTTCCTGTCTGCATCC |
| β-Actin | GAAGAGCTACGAGCTGCCTGA  CAGACAGCACTGTGTTGGCG |
| ANRIL | TACATCCGTCACCTGACACG  ACGAGGGGAGCCAGGAATAA |
| mus-ANRIL | CTTGTTGCGCTTCTCCCAAG  CCCTGGAGCCATCTTGTAGC |
| mus-β-actin | AAGACGAGGAGGAACTGAAC  CAAATCGGA CAACAAGACG- |
| mus-eNOS | AAGTGGGCAGCATCACCTAC  CCAAGCAGCGTCTTGAGGTA |
| mus-VCAM-1 | CTGGGAAGCTGGAACGAAGT  GCCAAACACTTGACCGTGAC |
| mus-vWF | CTGCACAGAAAAGCCCCGTG  AGGCTCATTCTCTTGCCATCT |
| mus-Drp-1 | CGTAAAAGGTTGCCCGTGAC  CACAGGCATCAGCAAAGTCG |
| mus-Mfn2 | AGAGGCAGTTTGAGGAGTGC  ATGATGAGACGAACGGCCTC |
| Mus-BDNF | ATTAGCGAGTGGGTCACAGC  ATTGCGAGTTCCAGTGCCTT |
