# Supplementary figures and images for: LncRNA ANRIL mediates endothelial dysfunction through BDNF downregulation in chronic kidney disease
Source: Cell Death Dis. 2022 Jul 29;13(7):661. doi: 10.1038/s41419-022-05068-1 (PMC9338026; doi:10.1038/s41419-022-05068-1)

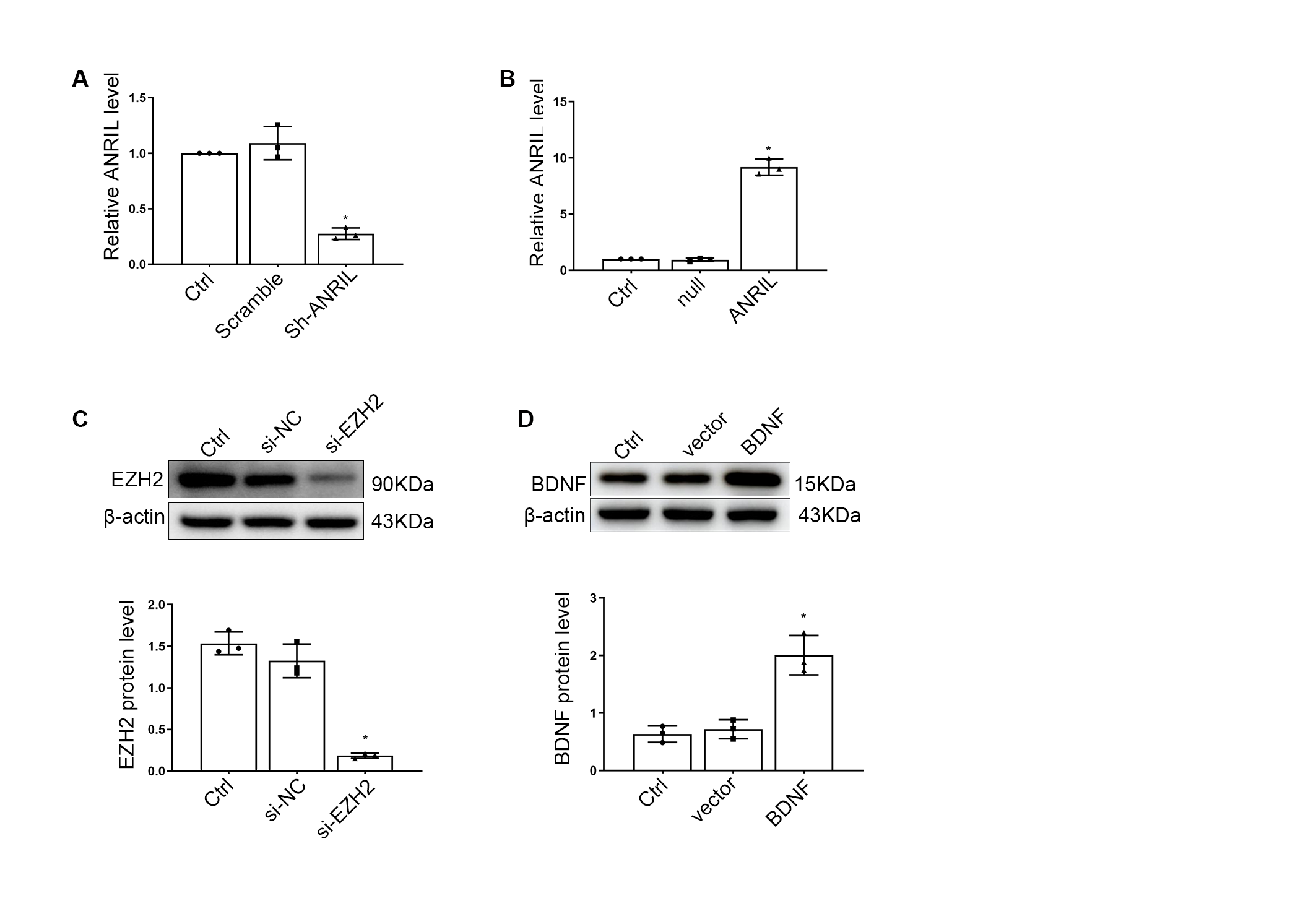

Supplement: Supplementary file 5 — Supplementary Figure. [file 41419_2022_5068_MOESM5_ESM.tif]

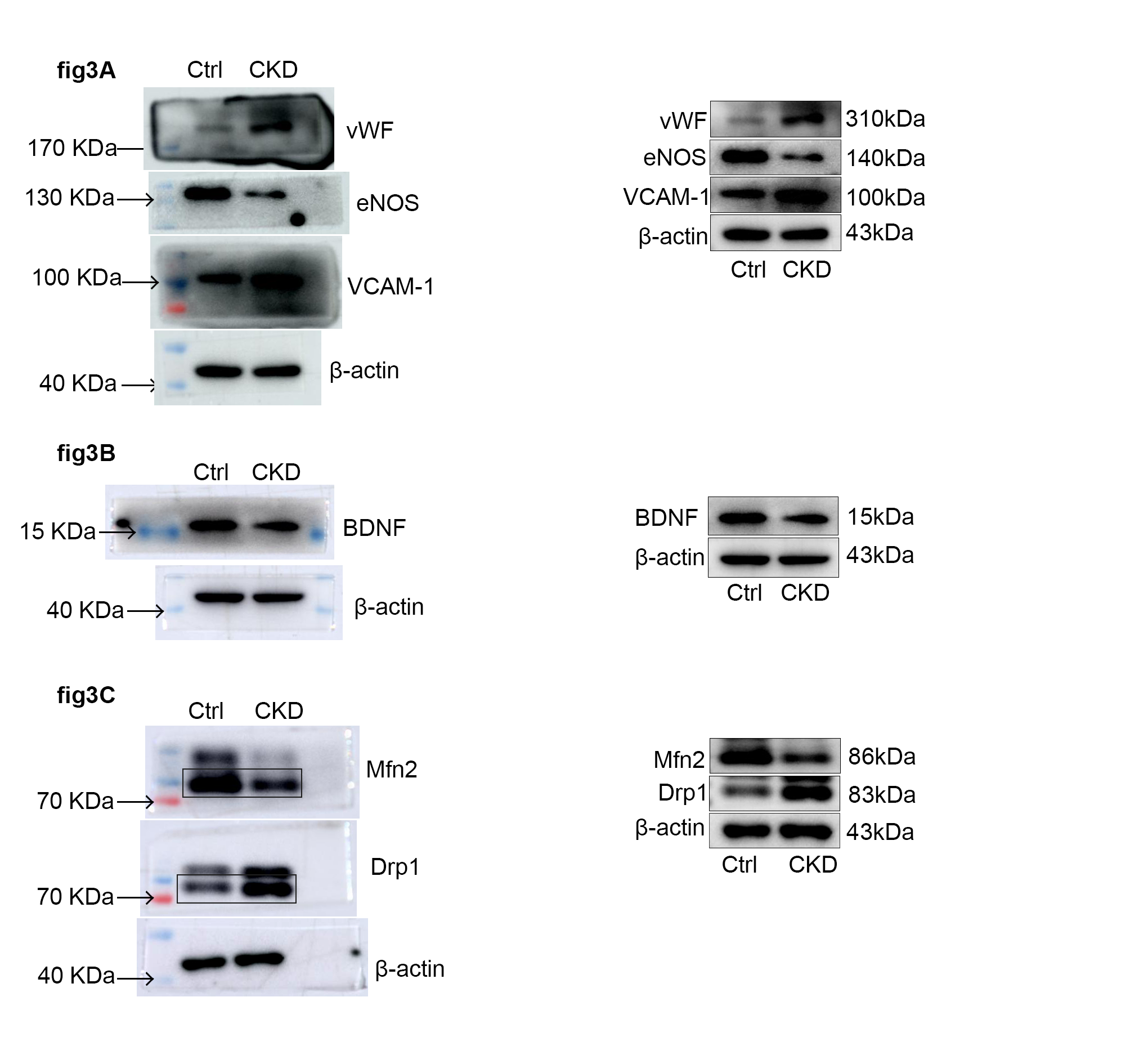

Supplement: Supplementary file 7 — Original western blots [file 41419_2022_5068_MOESM7_ESM.tif]

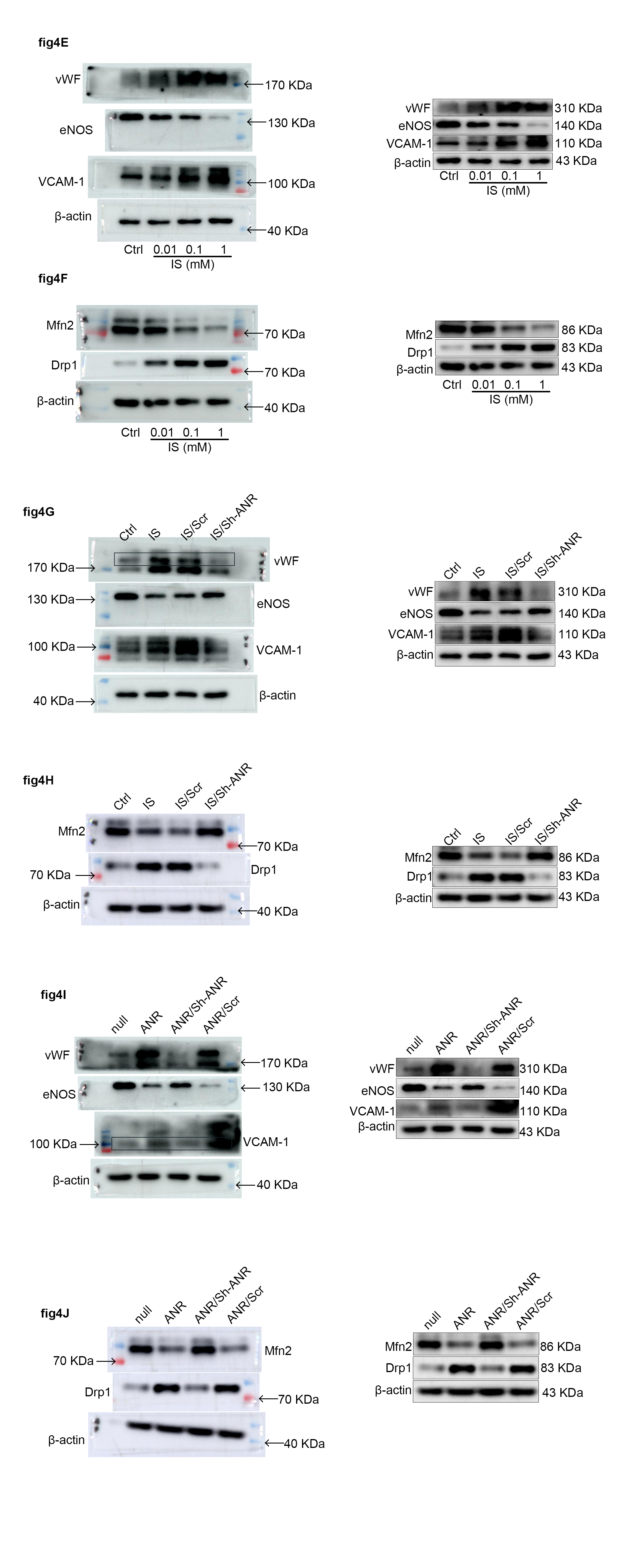

Supplement: Supplementary file 8 — Original western blots [file 41419_2022_5068_MOESM8_ESM.tif]

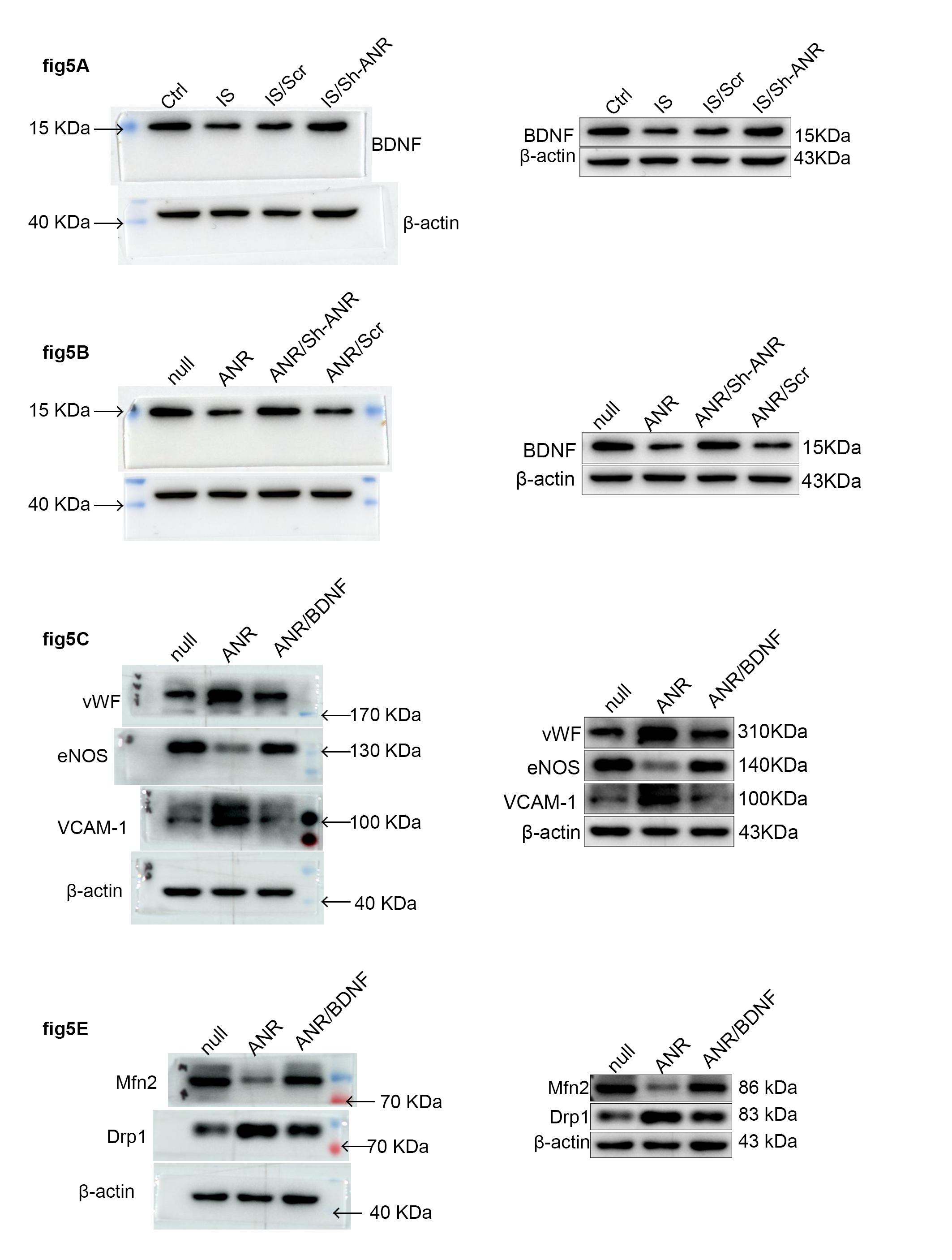

Supplement: Supplementary file 9 — Original western blots [file 41419_2022_5068_MOESM9_ESM.tif]

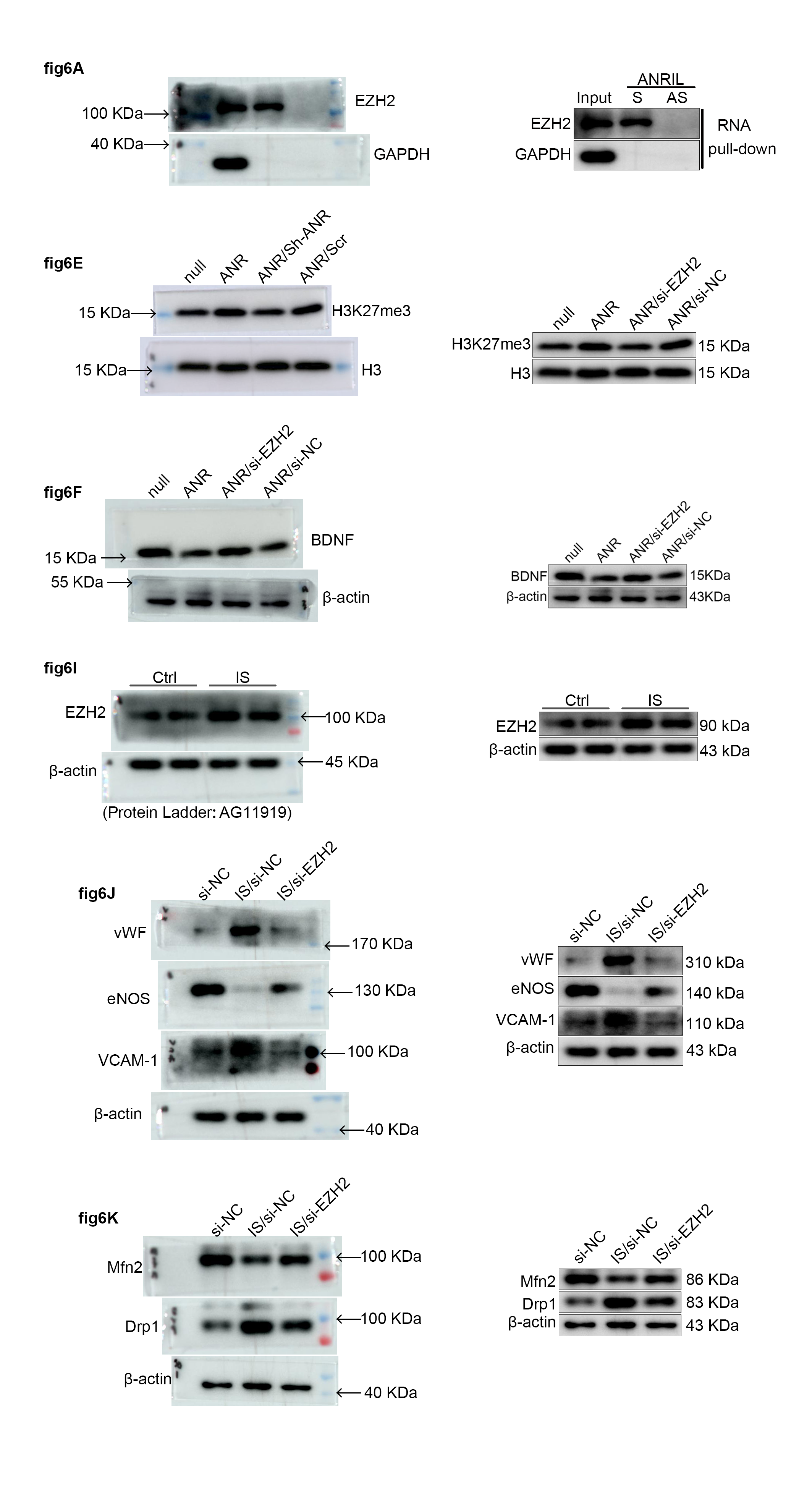

Supplement: Supplementary file 10 — Original western blots [file 41419_2022_5068_MOESM10_ESM.tif]

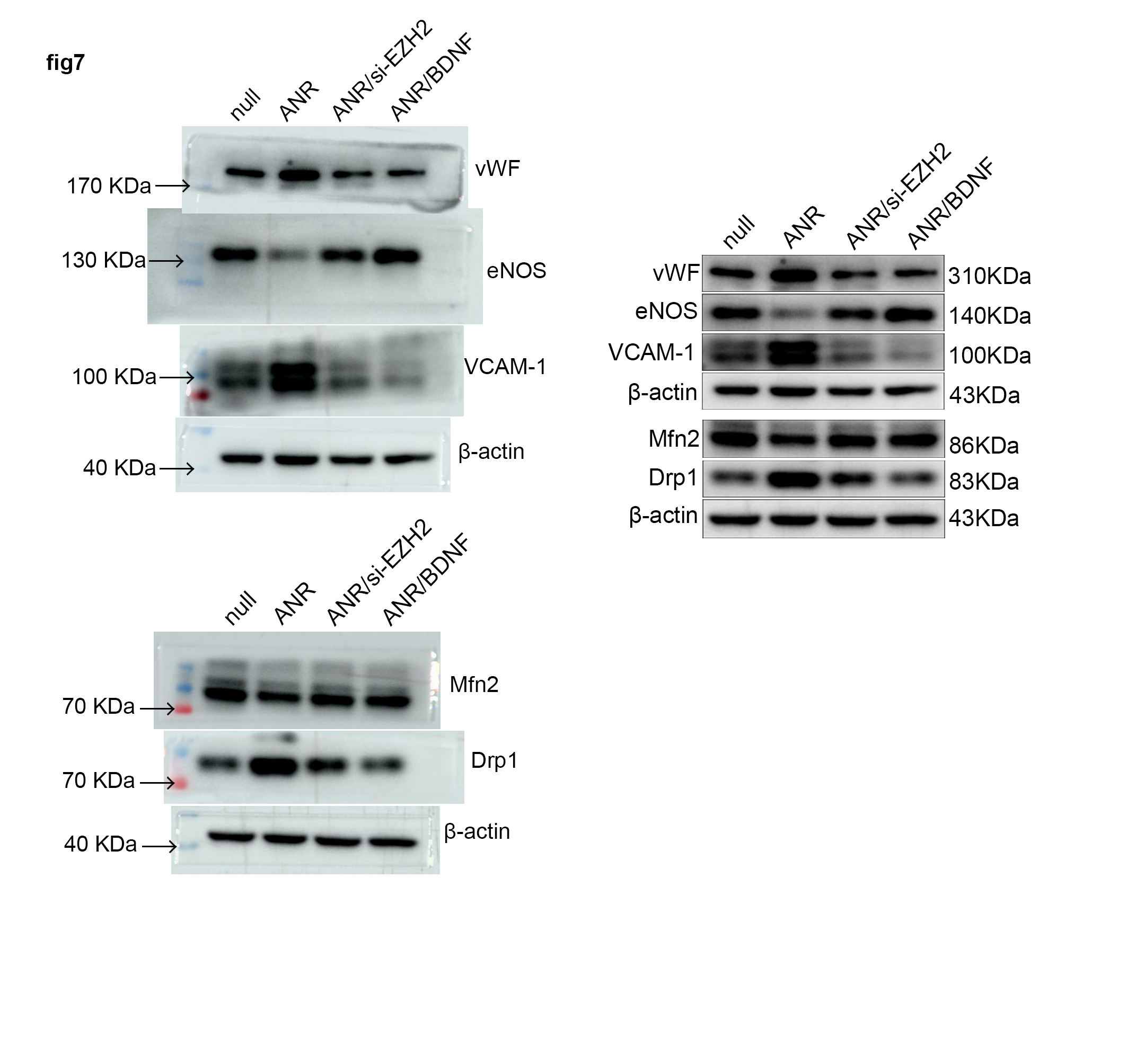

Supplement: Supplementary file 11 — Original western blots [file 41419_2022_5068_MOESM11_ESM.tif]

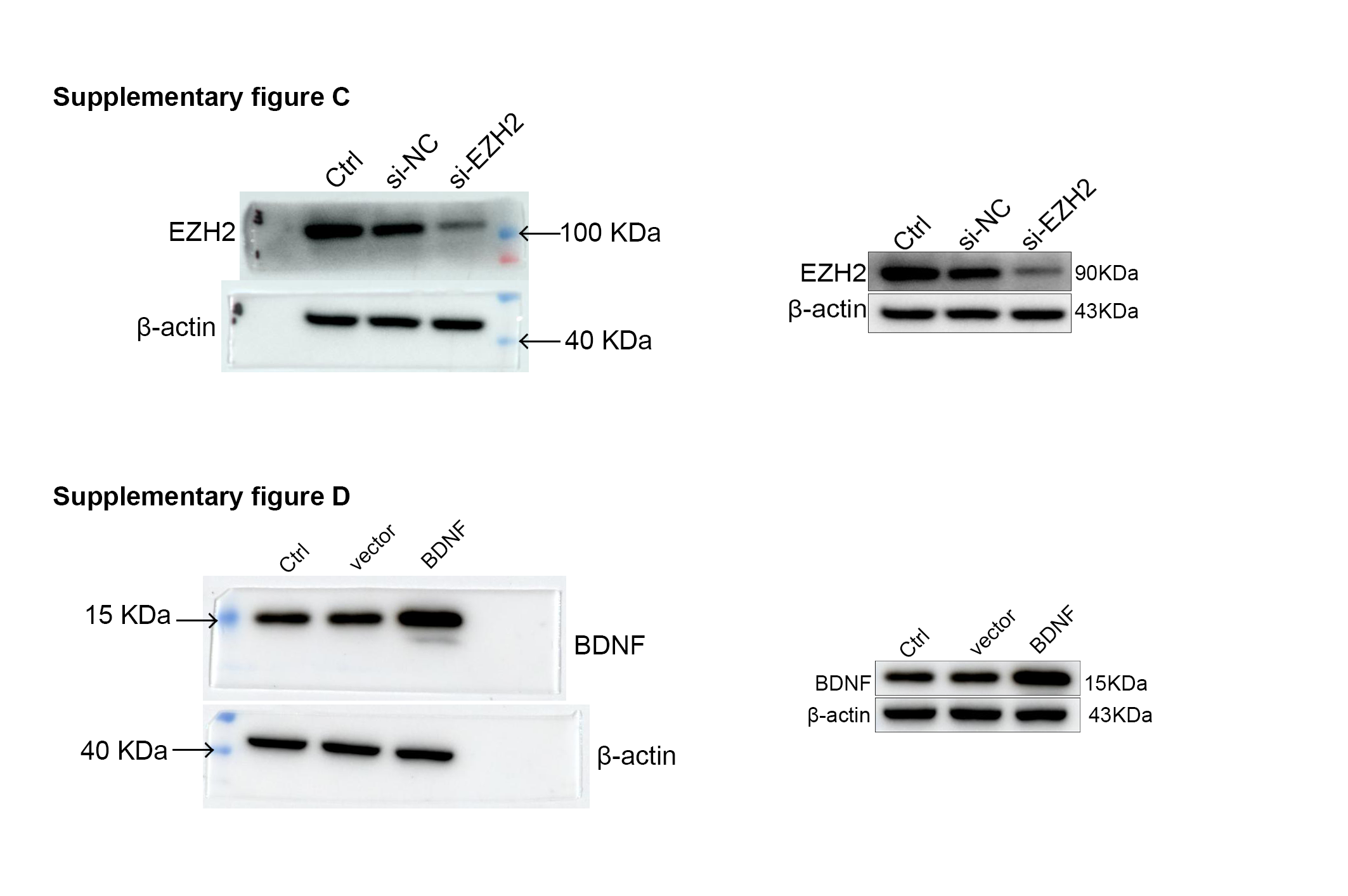

Supplement: Supplementary file 12 — Original western blots [file 41419_2022_5068_MOESM12_ESM.tif]
