# Supplementary material for: LncRNA ANRIL mediates endothelial dysfunction through BDNF downregulation in chronic kidney disease
Source: Cell Death Dis. 2022 Jul 29;13(7):661. doi: 10.1038/s41419-022-05068-1 (PMC9338026; doi:10.1038/s41419-022-05068-1)
Supplement: Supplementary file 6 — Supplementary Figure Legend [file 41419_2022_5068_MOESM6_ESM.docx]

**Supplementary Figure. Efficiency of lentivirus, siRNA and plasmid. (A, B)** Relative ANRIL expression in HUVECs were detected by realtime PCR. **(C)** The knockdown efficiency of EZH2 was confirmed by western bolt. **(D)** The overexpression efficiency of BDNF was confirmed by western bolt. Data were shown as mean ± SD (n = 3). (one-way ANOVA and the LSD test. ^*^P<0.05 versus respective control).
